# PCB Electronic Component Soldering Defect Detection Using YOLO11 Improved by Retention Block and Neck Structure

**DOI:** 10.3390/s25113550

**Published:** 2025-06-04

**Authors:** Youzhi Xu, Hao Wu, Yulong Liu, Xing Zhang

**Affiliations:** School of Mechanical Engineering, Anhui University of Technology, Ma’anshan 243002, China; huoxula@163.com (Y.X.); dulyam@163.com (Y.L.)

**Keywords:** PCB, defect detection, YOLO11n, RetBlock, MAFPN

## Abstract

Printed circuit board (PCB) assembly, on the basis of surface mount electronic component welding, is one of the most important electronic assembly processes, and its defect detection is also an important part of industrial generation. The traditional two-stage target detection algorithm model has a large number of parameters and the runtime is too long. The single-stage target detection algorithm has a faster running time, but the detection accuracy needs to be improved. To solve this problem, we innovated and modified the YOLO11n model. Firstly, we used the Retention Block (RetBlock) to improve the C3K2 module in the backbone, creating the RetC3K2 module, which makes up for the limitation of the original module’s limited, purely convolutional local receptive field. Secondly, the neck structure of the original model network is fused with a Multi-Branch Auxiliary Feature Pyramid Network (MAFPN) structure and turned into a multi-branch auxiliary neck network, which enhances the model’s ability to fuse multiple scaled characteristics and conveys diverse information about the gradient for the output layer. The improved YOLO11n model improves its mAP50 by 0.023 (2.5%) and mAP75 by 0.026 (2.8%) in comparison with the primitive model network, and detection precision is significantly improved, proving the superiority of our proposed approach.

## 1. Introduction

With the fast growth of the electronic information industry, printed circuit boards (PCB) are more and more widely used in various types of electronic equipment. For PCB assembly, due to the need to install various types of electronic components, surface mount technology (SMT) has become an important electronic assembly technology [1]. SMT replaces traditional plug-in technology and offers the advantages of lower assembly volume and weight, high density, and high reliability. However, due to instability in SMT welding processes such as reflow soldering or wave soldering, the welding of electronic components can produce random defects, such as missing electronic components, offsets, tombstoning, etc. If these defects are not detected, they will greatly impact PCB performance. Therefore, in actual production, surface solder defect detection in the electrical components of PCBs has become a key issue [2,3,4].

Early approaches to soldering defect detection in PCB electronic components included the traditional artificial visual detection method and automatic optical inspection method based on machine vision (AOI). The artificial visual inspection method mainly relies on a large number of artificial, lengthy visual inspections of PCB electronic components after SMT patch welding appearance. Although this method is simple, it is not only time-consuming and laborious due to the heavy inspection workload but also greatly limited by factors such as human eyesight, inspection experience, and emotions, with limited inspection efficiency and accuracy [5]. The AOI system makes use of a high-resolution camera to capture PCB sample surface images and detects defects such as missing electronic components, offsets, and tombstoning through image processing techniques. The AOI system detects defects mainly by distinguishing between the differences between the shape, color, and other features of the sample acquisition image and the defect-free template image [6]. However, this method can lead the system to incorrectly find defects due to small differences in the soldering color and shape of electronic components between the sample acquisition picture and the flawless template picture. In addition, the AOI system also requires very good cameras and light environmental conditions, which are costly, and the accuracy of the inspection results is also affected by the quality of the camera and the environmental conditions.

Deep learning algorithms have become popular in recent years, and their application in the defect detection domain has matured. At present, there are two main types of deep learning detection algorithms in the area of PCB electronic component soldering defects, namely, two-stage target detection algorithms based on candidate regions and the single-stage object detection approach for direct localization-based classification. Typical representatives of two-stage target detection algorithms are R-CNN [7], Faster R-CNN [8,9], Mask R-CNN [10,11], etc. First, these algorithms pick out pre-selected regions from the sample image, and then they classify and regress the pre-selected regions to filter out the target region. The advantage of this is that the inspection is more precise, but the amount of computation becomes larger and the model runs slower. The single-stage target detection algorithm is dominated by the YOLO algorithm [12], which does not need to produce pre-selected regions but directs the localization and classification of target objects. Its advantages are that the model’s calculations are small and run quickly, but the detection accuracy is not high. For PCB electronic part soldering, which involves a target dataset and detecting many electronic component targets against a complex background, the above methods do not give good results and are not well-suited to industrial production. Therefore, in this paper, we have improved and innovated the YOLO11n model [13] so that it can be better applied to PCB electronic component welding defect dataset samples. We improved the C3K2 module fusion Retention Block (RetBlock) [14] in the YOLO11n backbone network, forming the RetC3K2 module, which is more suitable for detecting sample datasets with complex backgrounds. In addition, we also improved the original neck network fusion Multi-Branch Auxiliary FPN structure [15] of YOLO11n, forming a multi-branch auxiliary neck network structure that enhances sensitivity to small target detection and reduces leakage rate. Based on our proposed improved method of YOLO11n, all surface patch element solder defects of varying sizes can be detected within a single image, which improves detection accuracy compared to the original network model. 

Our research contributions in this paper are mainly in the following areas:

1. Unlike earlier detection approaches that only detect soldering defects on a single PCB surface mount component, we use an innovative and improved model structure based on YOLO11n for the first time to detect soldering defects on a variety of surface patch components of different sizes and shapes within a single picture.
2. We improve the C3K2 module in the YOLO11n backbone network model to RetC3K2 module, which utilizes the combination of C3K2 module and RetBlock. This modification retains the attention mechanism to strengthen the overall modeling ability, make up for the limitation of pure convolution, and improve the detection accuracy.
3. We improve the original neck network of YOLO11n into a multi-branch auxiliary neck network structure, so that the shallow information is retained as an auxiliary branch into the deep network, which enhances the model’s multi-scale feature fusion capability and improves the accuracy of detecting defects on small targets.

This paper is organized as follows. In Section 2, we analyze the development of the research area of soldering defect detection in PCB electronic components. Section 3 describes the knowledge of theoretical principles of our research approach. Section 4 conducts a comparative analysis of experiments with different previous approaches. Section 5 briefly summarizes the entire article.

## 2. Related Work

Defect detection for soldering PCB electronic components is a crucial process in the electronic assembly industry. The current mainstream defect detection approaches are broadly categorized into two types: early traditional defect detection and recognition algorithms and deep learning-based defect detection and recognition algorithms.

Early traditional defect detection algorithms primarily combine machine learning with digital image processing techniques [16]. Jiang et al. [17] proposed a novel method on PCB solder paste defect detection, using bionic color features to characterize the solder paint image and introducing an innovative sub-flow shape learning approach. This method effectively identifies the poor-quality solder paste while addressing the limitations of the traditional method, such as high cost and slow detection speed. Wu [18] achieved high accuracy in PCB defect detection by extracting color and geometric features of solder joints and subsequently applying the random forest approach to classify and detect the defects. Luo et al. [19] proposed a novel multistep preprocessing method on the basis of MiniLED backlight PCB pad images to improve the accuracy. Their approach incorporated threshold segmentation, fuzzy C-mean clustering-based segmentation, and edge detection based on Canny operator, among other techniques. However, the threshold range selected for threshold segmentation requires greater precision, and inappropriate thresholds may cause problems such as under-segmentation or over-segmentation. Zhu et al. [20] presented a detection method employing wavelet de-noising technology. The detection accuracy is further enhanced by wavelet de-noising and histogram equilibrium enhancement technologies to achieve the detection percentage of defects to 100% and the recognition percentage to more than 90%. Despite the mentioned machine learning-based detection methods having achieved satisfactory results, they exhibit several limitations. First, feature extraction in the case of complex target samples is time-consuming and labor-intensive, and there is a lack of generalization ability. Second, these algorithms are typically limited to classification and identification tasks, and additional postprocessing is required for localization of defects.

Recently, deep learning-based detection approaches have been extensively used in PCB defect detection. Ding [21] enhanced the Faster R-CNN network framework and proposed a specialized detection framework for small defects, significantly improving the efficiency of PCB defect detection for complex and diverse PCB defects. Experimental results demonstrate that the method performs well on public datasets and has good generalization capability. Liu et al. [22] improved the Cascade Mask R-CNN defect detection framework by replacing its original backbone with a Swin-Transformer network to enhance the defect feature acquisition quality from samples, thereby increasing the detection accuracy of the soldering defects on the PCB surface patches. Nevertheless, the introduction of a Swin-Transformer network results in higher computational costs and a longer training and inference time. Li [23] proposed a novel deep integration approach for PCB solder defect detection, which mainly combines YOLOv2 and Faster R-CNN to increase the detection percentage as well as decrease the false alarm percentage. Du et al. [24] improved the YOLOv5 network by introducing a bi-directional feature pyramid network (BiFPN) and convolutional block attention module (CBAM) to enhance the multi-scale feature fusion and realize the goal of increasing the precision and real-time performance. Chen [25] developed a Transformer-YOLO model detection approach, which combined the Swin-Transformer and YOLOv5 to optimize the acquisition of feature images and improve the accuracy and efficiency of PCB defect detection. Liu [26] presented a CSYOLOv8 model based on YOLOv8, which enhances detection accuracy by designing a composite backbone structural network for additional feature representation to strengthen the feature expression capability. Zheng [27] proposed an FDDC-YOLOv10 network on the basis of YOLOv10 network model, incorporating both the full-dimensional dynamic convolution module (FDDC) and the cross-channel enhanced attention (CECA) block. This design strengthens the ability of feature extraction and the local mutuality between channels, significantly improving the detection capability of small target defects. The main advantage of the single-stage target detection algorithm based on the YOLO model algorithm for PCB defect detection is that its model calculation is small and inference speed is fast. But the shortcoming is that the detection accuracy needs to be improved.

## 3. Proposed Method

First, we perform a dataset expansion experiment on the PCB surface patch component soldering defects experimental dataset using the ControlNet-based [28] stable diffusion model [29]. Subsequently, we employ the improved YOLO11n network model to detect the expanded dataset. The overall improved network model still consists of the backbone network, the neck network, and the detection head. The C3K2 block in the backbone network is improved to RetC3K2 block using Retention Block, and the original neck network is improved to a multi-branch auxiliary neck network structure.

### 3.1. Expanding Dataset Using ControlNet-Based Stable Diffusion Models

**Figure 1:**
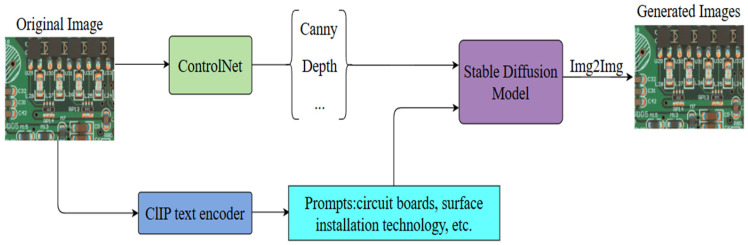
The structure of ControlNet-based stable diffusion model.

**Figure 2:**
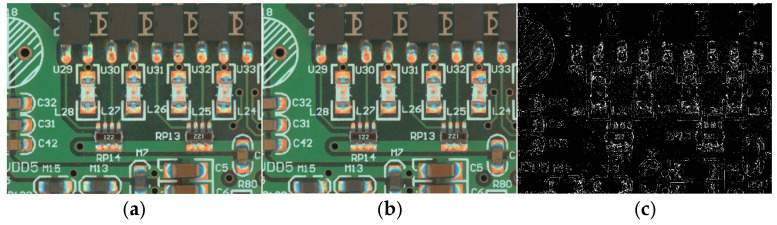
Sample generation results: (**a**) original image; (**b**) generated image; (**c**) difference between the original image and the generated image.

As the PCB surface patch component soldering defects dataset has less variety in actual production, which is not enough to support deep learning model training, it needs to be expanded. The commonly used expansion method is to use the adversarial generative network (GAN) [30] for expansion, but this experimental dataset is a non-independent sample, there will be more than one surface patch component target in the sample image, which will contain both normal and defective component welding detection targets, and the adversarial generative network cannot be applied to expand the dataset. In this experiment, the ControlNet-based stable diffusion model is used to expand the dataset, and its structure is shown in Figure 1 below. Firstly, Contrastive Language-Image Pre-training (CLIP) [31] is utilized to extract sample key cues from the input sample images. The textual cues are then encoded and input into the latent space, where they are fused with the features in the latent space to further control the model output. The cue words are selected to be re-injected into the stable diffusion model to assist the cueing of Img2Img with the higher-frequency cue words. At the same time, ControlNet is utilized to extract key features from the input image and feed them to the stable diffusion model for additional control over the output image. Ultimately, the stable diffusion model generates an expanded sample image that closely resembles the original input image with respect to its logical distribution and spatial layout, which are visually very similar to each other but very different at the pixel level. As illustrated in Figure 2 below, Figure 2a and Figure 2b show the input sample image and generated sample image, respectively, while Figure 2c represents the pixel-level differences between them. In addition, the generated image does not need to be re-labeled with an annotation tool, and its label information can be shared with the original image, significantly reducing time and labor costs.

### 3.2. Improved Overall Network Architecture of YOLO11n

**Figure 3:**
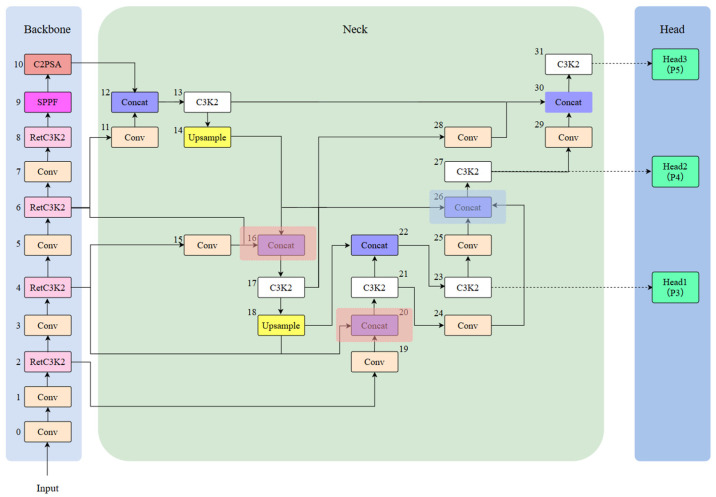
Diagram of the overall network architecture of the improved YOLO11n.

The complete framework of the improved YOLO11n is illustrated in Figure 3 below. As the experimental results have shown, the YOLO11n “https://docs.ultralytics.com/zh/models/yolo11” (accessed on 10 February 2025) detection network exhibits limited performance in the detection of PCB surface patch electronic component soldering defects, such as the detection of a variety of target types, different scales, and the more complex background dataset detection performance is insufficiently sensitive to small target detection and it is easy to miss the detection. To address these limitations, we improve the C3K2 module in the backbone combined with Retention Block to RetC3K2 module. Additionally, we improve the original neck network to a multi-branch auxiliary neck network.

The process principle of the model to detect defects is mainly to preprocess the input image first, such as scaling and normalization, and then carry out feature extraction through the backbone network. These extracted features are subsequently fed into the neck network for multi-scale feature fusion. At the end, the detection head is in charge of outputting the ultimate prediction for both target detection and classification.

### 3.3. RetC3K2 Module Structure Theory

**Figure 4:**
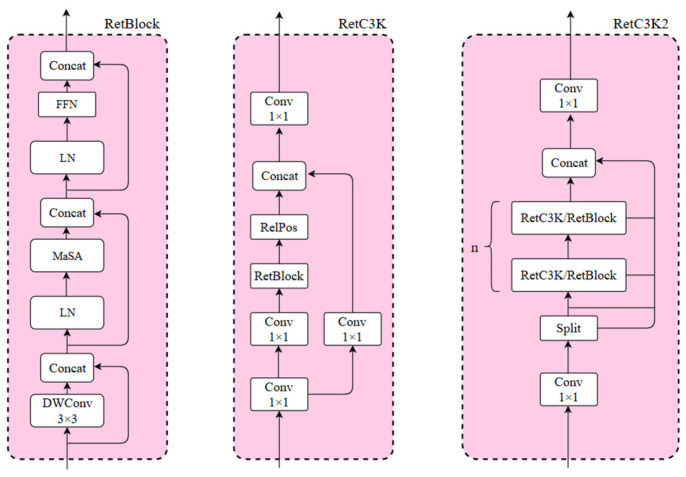
RetBlock, RetC3K, and RetC3K2 module architecture schematics.

The C3K2 module is an important and efficient feature extraction component in the YOLO11n model, which is improved on the basis of the design structure of the traditional C3 module by introducing multi-scale convolutional kernels and adopting a channel separation strategy, enabling the model to capture contextual information in a larger range and enhances the ability of feature extraction in complex scenarios and deep-level tasks. However, the original C3K2 module relies solely on a purely convolutional stacking structure, which is computationally efficient and stable for training, but it lacks flexibility, with branches fixed to bottleneck layers and limited receptive fields, and is unable to dynamically adjust the receptive fields. Its localization limitation is also large and lacks global dependency modeling capability. Furthermore, the original C3K2 module performs poorly when processing datasets like PCB surface patch component soldering defects, which are detected with smaller targets, more target types, and complex and dense backgrounds. To address this issue, we introduce Retention Block (RetBlock) into the C3K2 module, upgrading it to the RetC3K2 module. RetBlock introduces dynamic retention of attention based on the local perception of traditional convolution. Through the coordinated design of convolution and attention, local–global feature cooperative modeling is achieved and the accuracy of small target detection is improved. RetBlock, with its structure illustrated in Figure 4, first employs Depthwise Separable Positional Convolution (DWConv) to inject local positional information, thereby enhancing the model network’s ability for the recognition of low-level feature details. Subsequently, Manhattan Self-Attention (MaSA) [14], as the core component of RetNet, mainly utilizes the dynamic spatial attention mechanism to strengthen the ability of the model to capture the global context and improve the accuracy of small target detection. Finally, the nonlinear transformation is introduced through a feed-forward network (FFN) to further enhance feature representation.

The structure of the RetC3K2 module, obtained by combining the C3K2 module with the RetBlock, is illustrated in Figure 4. Firstly, the numbers of channels are adapted through convolution and then split into two branches using the Split operation. One branch retains the original features as the residual benchmark, while the other branch connects to the module components in series by RetC3K or RetBlock, and the number of its series n is generally chosen as 2. The parameter controlling whether the branch connects to the module components via RetC3K or RetBlock is C3K, which balances the computational cost and performance. When C3K is true, the model chooses to pass the RetC3K module, whose internal relative position encoding RelPos based on Manhattan distance generates the attenuation mask, enabling the model to adaptively learn to adjust the weights according to the correlation of different positions, realizing a kind of spatial a priori perception, and the integration of MaSA allows the model to understand the spatial relationship between pixels better. When C3K is false, the model chooses to pass the RetBlock module. In this case, it needs to pass RelPos, encoding the relative position, from the outside at the meantime to keep the representation of the spatial relationship and the integrity of information.

Compared with the original C3K2 module, RetC3K2 introduces RetBlock, which replaces the local receptive field of pure convolution with the MaSA attention mechanism, and, at the same time, combines with the attenuation mask generated by the relative position encoding RelPos, which is different from the original convolutional kernel with fixed weights, and allows the model to adaptively learn the importance of different positions. This enhancement significantly improves the detection accuracy of target defects.

### 3.4. Multi-Branch Auxiliary Neck Network

The neck network structure of the original YOLO11 primarily employs a Path Aggregation Network (PAN), which combines and splices feature maps from different layers through an up-sampling module and then performs feature fusion through a convolution module. The introduced C3K2 module in this structure helps address the multiscale feature fusion challenges in target detection. However, this approach treats all feature maps equally during fusing, lacking targeted enhancement for critical features. In complex scenes, low-quality features may be fused indiscriminately, leading to an increase in the false detection rate. Moreover, the feature reuse efficiency is low, and features lack an adaptive filtering mechanism when passing across layers, which in turn leads to shallow detailed features (e.g., P3) being easily overwhelmed by high-level semantic features (e.g., P5) when passing to the deeper layers, affecting the accuracy for small target detection.

**Figure 5:**
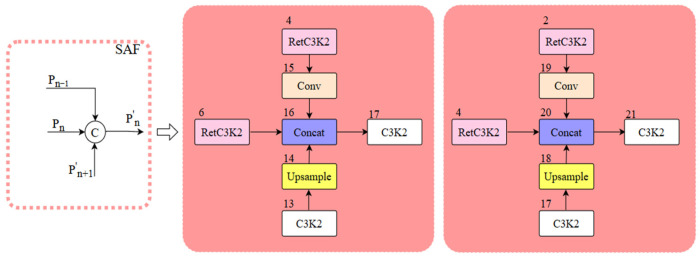
SAF structure schematic.

**Figure 6:**
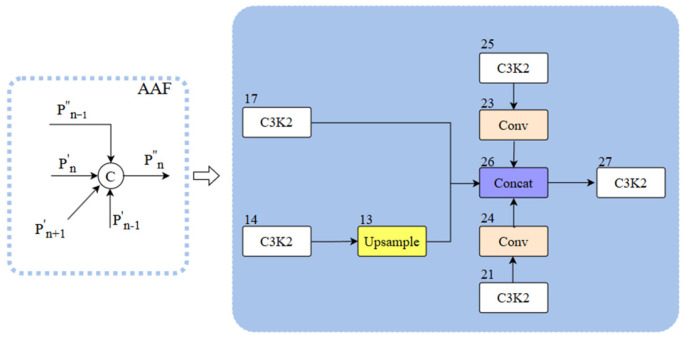
AAF structure schematic.

To address the aforementioned issues, we integrate the original neck network with the Multi-Branch Auxiliary FPN (MAFPN) structure and propose the multi-branch auxiliary neck network structure. The MAFPN primarily consists of Surface Auxiliary Fusion (SAF) and Advanced Auxiliary Fusion (AAF). The SAF structure diagram is illustrated in Figure 5, where P_n−1_, P_n_, and P_n+1_ mean feature maps with various resolutions; P_n_ represents the feature layer of the backbone structure, and P′_n_ and P″_n_ denote the two paths of the MAFPN. We integrated the SAF structure into the two Concat modules (numbered 16 and 20, indicated by light red boxes in Figure 3). The main role of the fused SAF structure is to integrate the features of the deep layer information with the same-layer and high-resolution shallow layer within the backbone structure with each other through cross-layer hopping connections and to retain the shallow layer information as an auxiliary branch into the deeper network to enhance the performance of detection for a small target. The AAF structure is illustrated in Figure 6, which combines the aggregated layers across the shallow high-resolution layer P′_n+1_, the superficial low-resolution layer P′_n−1_, the similar superficial layer P′_n_, and the previous layer P″_n−1_ that are integrated with each other to produce output P″_n_. We fuse the AAF structure into the Concat module (number 26, indicated by the blue box in Figure 3). The fused AAF structure can simultaneously merge feature information from four different layers, enabling the output layer to retain comprehensive multi-scale information and thereby improving the performance of medium-sized target detection.

The multi-branch auxiliary network structure, formed by integrating the original neck network with the MAFPN structure, relies on the SAF, which can consolidate the output features of the backbone and the neck, thus preserving the shallow information in deeper networks. At the same time, relying on the deeper AAF can integrate the multi-scale feature information and deliver diverse gradient information to the output layer. This architecture significantly improves the detection accuracy of defects for a small target.

## 4. Results of the Experiment

### 4.1. Experimental Dataset and Related Parameter Settings

**Figure 7:**
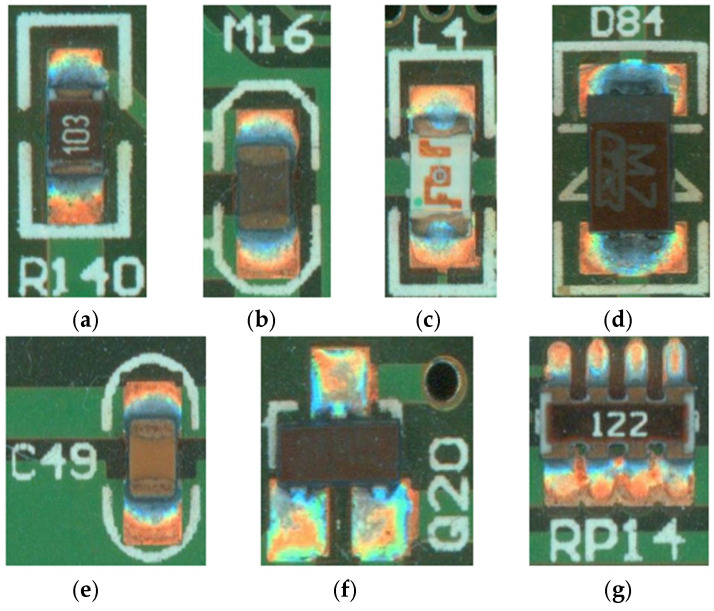
Images of mounted electronic components: (**a**) resistor; (**b**) thermistor; (**c**) inductor; (**d**) diode; (**e**) capacitor; (**f**) triode; and (**g**) potentiometer.

The dataset utilized in our experiment is the PCB surface mount electronic component soldering defects, containing a total of 210 samples. These samples are divided into the training, validation, and test dataset according to the proportions of 8:1:1. It mainly contains seven kinds of patch electronic components, and the schematic diagram is shown in Figure 7. Each SMT electronic component has corresponding defect types: capacitance and resistance missing, offset, and tombstoning, inductance missing as well as offset, and diodes, transistors, thermistors, and voltage dividers missing; examples are shown in Table 1 below. The normal plus defective labels add up to a total of 19 distinct labeled categories.

Our experiments were conducted on a Linux system equipped with an NVIDIA GeForce RTX 4060Ti GPU. The deep learning frameworks consisted of CUDA12.4, torch-2.5.1, and Python-3.9.21. We adopted the following training parameters: a learning rate of 0.001, batch size of 2, and 300 training epochs, using the Stochastic Gradient Descent (SGD) optimizer.

### 4.2. Comparative Analysis of Experimental Results

The methodology of this defect detection experiment is based on the improved YOLO11n defect detection algorithm, by comparing the two-stage detection algorithms Mask R-CNN, Swin Transformer Mask-CNN, and Cascade Mask R-CNN algorithms based on ConvNeXT with the same single-stage detection algorithms YOLO11n, to demonstrate the superiority of our improved algorithm on the PCB surface patch component soldering dataset. The experimental metrics utilized in our experiment are precision (P), average recall (AR) and mean average precision (mAP) of the detection tasks under various IoUs as the main metrics to validate the superiority of our method. The metrics of the counts of model parameters (Params) and Giga Floating-Point Operations Per Second (GFLOPs) are also utilized to assess the computational cost of the model.

Table 2 presents the comparison between our improved YOLO11n defect detection algorithm and the two-stage detection algorithm. It can be seen that our proposed method does not have as high mAP as well as AR accuracy metrics as the ConvNeXT-based Cascade Mask R-CNN algorithm, but the number of Params and GFLOPs of our proposed method are much smaller in comparison, which is not very demanding on computers, and the computational complexity is very small.

Table 3 displays the comparison between the improved YOLO11n defect detection algorithm and the original YOLO11n algorithm, as well as single-stage detection algorithms such as YOLOv10n and YOLOv8n. It can be observed that the average accuracy of our improved YOLO11n algorithm has been greatly improved compared to the original YOLO11n model, with its mAP50 improved from the original 0.927 to 0.950, an improvement of 0.023 (2.5%), and its mAP75 improved from the original 0.924 to 0.950, an improvement of 0.026 (2.8%). The improved model precision P and average recall AR are also improved by 0.66% and 0.67%, respectively. The Params and GFlops metrics are comparable to the original network model, with the same computational cost. The quantitative results clearly demonstrate the superiority of our proposed improvements.

Table 4 presents the ablation study results comparing different module combinations in the improved YOLO11n architecture. From the table, it can be found that YOLO11n combined with the RetC3K2 module and multi-branch assisted necking network has a good improvement in mAP compared with the single module, and the Params as well as GFlops indexes are not much different and the computational cost is about the same. These results validate the superiority of the two improved methods after superposition.

**Figure 8:**
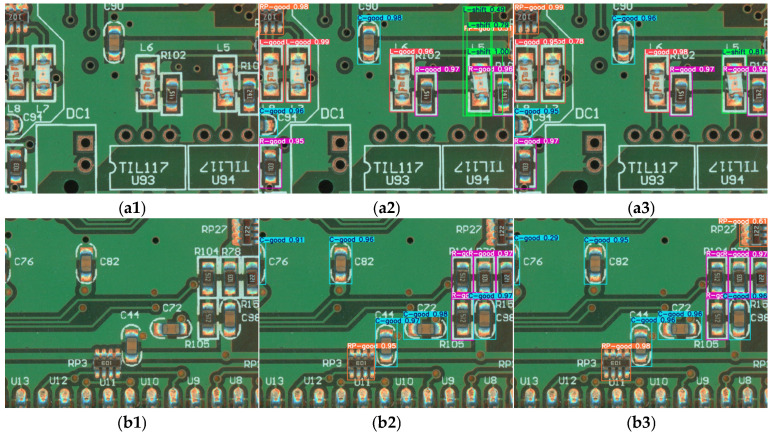

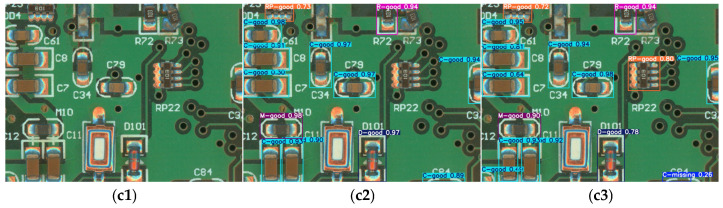
Partial image of defect detection results: (**a1**–**a3**) the original images of defect detection; (**b1**–**b3**) original YOLO11n model detection results; (**c1**–**c3**) the improved YOLO11n model detection results.

In Figure 8 we list some soldering defect detection results of PCB surface mount electronic components with the improved YOLO11n model and the original YOLO11n model; Figure 8(a1–a3) show the original picture of our experimental test, Figure 8(b1–b3) show the original YOLO11n model’s detection results on the test images, and Figure 8(c1–c3) show the detection results of the improved YOLO11n model on the test images. It can be intuitively found that the original YOLO11n model is prone to misdetection or missed detection in the face of the complex detection background based on multiple detection objects, such as the misdetection of the offset inductance as multiple targets in Figure 8(c1), the missed detection of the voltage divider in the upper-right corner of Figure 8(c2), and the missed detection of the voltage divider in the middle of Figure 8(c3) due to the appearance of some complex holes in the background around it. Compared with our improved YOLO11n detection method, our method effectively addresses the above misdetection and missed detection while maintaining high detection accuracy.

## 5. Conclusions

This paper focuses on detecting soldering defects in PCB surface mount electronic components. To improve the detection accuracy of the original YOLO11n, we first enhance the C3K2 module in the backbone to the RetC3K2 module by using Retention Block (RetBlock). This upgraded module addresses the limited receptive field of conventional convolution operations through integration with the MaSA attention. Furthermore, we incorporate relative position encoding, RelPos, to generate an adaptive attenuation mask, replacing the original fixed-weight convolutional kernels. This modification enables the model to dynamically learn the significance of diverse positions, which greatly improves the detection performance of target defects. Then, we develop a multi-branch auxiliary neck network (MAAN) by integrating the Multi-Branch Auxiliary FPN (MAFPN) structure. The enhanced architecture effectively integrates the output features of the backbone and the neck while preserving the shallow information in the deep network, which strengthens the ability of integrating the multiscale feature information, and delivers diversified gradient information to the output layer, substantially improving the detection accuracy of defects in small targets.

The results of the experiments in our paper demonstrate the superiority of our improved YOLO11n model for soldering defect detection of PCB surface-mounted electronic components. Compared with the original YOLO11n model network, our model achieves significant improvements of 0.023 (2.5%) in mAP50 and 0.026 (2.8%) in mAP75, along with markedly enhanced detection accuracy. The proposed method also maintains high accuracy as well as detection productivity, presenting the practical solution for defect detection in industry. Although the whole performance of the improved model has been enhanced, there are still some deficiencies that need to be refined, and there is still potential for improvement on detection accuracy and detection precision. The future research direction will focus on exploring more advanced modeling algorithms to improve the detection accuracy, expand detectable target types, and enhance the model’s applicability in industrial settings.

## Figures and Tables

**Table 1:** PCB electronic component soldering defect schematic table.

Picture Examples	Name of Defect	Picture Examples	Name of Defect	Picture Examples	Name of Defect
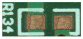	resistor missing (R-missing)	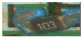	resistor shift (R-shift)	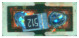	resistor tombstone (R-tombstone)
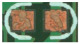	capacitor missing (C-missing)	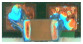	capacitor shift (C-shift)	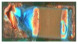	capacitor tombstone (C-tombstone)
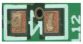	inductor missing (L-missing)	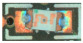	inductor shift (L-shift)	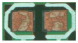	thermistor missing (M-missing)
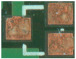	triode missing (Q-missing)	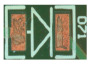	diode missing (D-missing)	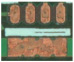	Potentiometer missing (RP-missing)

**Table 2:** Comparative analysis of improved YOLO11n detection algorithm and two-target phase detection algorithm.

Model	mAP	mAP50	mAP75	AR	Params/M	GFlops/G
Mask R-CNN [11]	0.746	0.934	0.929	0.814	43.75	258.2
ST–Mask R-CNN [32]	0.864	0.947	0.944	0.892	47.37	261.8
ConvNeXt Cascade Mask-RCNN [33]	0.886	0.962	0.962	0.907	85.84	472.3
Our proposed method	0.850	0.950	0.950	0.899	2.58	6.9

**Table 3:** Comparative analysis of improved YOLO11n detection algorithm and single-target phase detection algorithm.

Model	P	mAP50	mAP75	AR	Params/M	GFlops/G
YOLOv8n	0.886	0.909	0.901	0.864	3.01	8.1
YOLOv10n	0.863	0.881	0.881	0.835	2.27	6.5
YOLO11n	0.898	0.927	0.924	0.893	2.59	6.3
Our proposed method	0.904	0.950	0.950	0.899	2.58	6.9

**Table 4:** Comparative data table of ablation experiments with improved YOLO11n.

Model	P	mAP50	mAP75	AR	Params/M	GFlops/G
YOLO11n	0.898	0.927	0.924	0.893	2.59	6.3
YOLO11n + RetC3K2	0.892	0.937	0.935	0.881	2.47	6.2
YOLO11n + MAFPN Neck	0.900	0.942	0.938	0.884	2.70	7.1
YOLO11n + RetC3K2 + MAFPN Neck	0.904	0.950	0.950	0.899	2.58	6.9

## Data Availability

The data that support the findings of this study are available from the corresponding author, H.W., upon reasonable request.
